# Superparamagnetic Artificial Cells PLGA-Fe_3_O_4_ Micro/Nanocapsules for Cancer Targeted Delivery

**DOI:** 10.3390/cancers15245807

**Published:** 2023-12-12

**Authors:** Tao Wang, Thomas Ming Swi Chang

**Affiliations:** Artificial Cells and Organs Research Centre, Departments of Medicine and Biomedical Engineering, Faculty of Medicine, McGill University, Montreal, QC H3G 1Y6, Canada

**Keywords:** superparamagnetic artificial cells, PLGA-Fe_3_O_4_ micro/nanocapsules, targeted cancer therapy, magnetic hyperthermia, photothermal therapy, targeted delivery

## Abstract

**Simple Summary:**

Cancer is the main cause of human death. Nanomedicine provides a new strategy for the treatment of diverse cancers. Among them, superparamagnetic artificial cell PLGA-Fe_3_O_4_ micro/nanocapsules demand great attention due to their excellent properties, such as targeted delivery, magnetic response, and biocompatibility. The aim of this review is to summarize the application of superparamagnetic artificial cells (PLGA-Fe_3_O_4_) micro/nanocapsules in cancer drug targeted delivery and their preparation methods, providing insights into the future development of superparamagnetic artificial cells (PLGA nano/microcapsules) for magnetic targeting in cancer treatment.

**Abstract:**

Artificial cells have been extensively used in many fields, such as nanomedicine, biotherapy, blood substitutes, drug delivery, enzyme/gene therapy, cancer therapy, and the COVID-19 vaccine. The unique properties of superparamagnetic Fe_3_O_4_ nanoparticles have contributed to increased interest in using superparamagnetic artificial cells (PLGA-Fe_3_O_4_ micro/nanocapsules) for targeted therapy. In this review, the preparation methods of Fe_3_O_4_ NPs and superparamagnetic artificial cell PLGA-drug-Fe_3_O_4_ micro/nanocapsules are discussed. This review also focuses on the recent progress of superparamagnetic PLGA-drug-Fe_3_O_4_ micro/nanocapsules as targeted therapeutics. We shall concentrate on the use of superparamagnetic artificial cells in the form of PLGA-drug-Fe_3_O_4_ nanocapsules for magnetic hyperthermia/photothermal therapy and cancer therapies, including lung breast cancer and glioblastoma.

## 1. Introduction

Cancer poses a severe threat to human health, and the rate of cancer incidence has been steadily increasing. In statistics compiled for 2020, an estimated 19.3 million new cancer cases (18.1 million excluding nonmelanoma skin cancer) and almost 10 million cancer deaths occurred around the world—close to one in three people during their lifetimes will be diagnosed with some type of tumor disease [1]. Nowadays, cancer treatment methods include surgical resection, radiation therapy, chemotherapy, and immunotherapy [2]. However, there are still three disadvantages for these methods: (1) it is difficult to completely remove the tumor cells by surgery [3], (2) the normal cells around the irradiated site are damaged to a certain extent while tumor cells are killed under radiotherapy [4], (3) traditional chemotherapeutic agents cannot achieve specific distribution in vivo, and they can simultaneously damage both tumor cells and normal cells [5]. Therefore, researchers are interested in the development of an improved targeting and efficient strategy for cancer treatment with minimal side effects.

In 1964, Chang published the first paper on artificial cells (semipermeable microcapsules) [6] that can be used for a number of medical applications [7,8], including drug delivery, enzyme/gene therapy, cancer therapy, cell/stem cell therapy, regenerative medicine, hemoperfusion, blood substitutes, encapsulated microbes [7,8,9,10,11,12], and others. Recent developments in artificial micro/nanomotors and COVID-19 vaccines can be found in recent publications [9,10,11,13,14]. In 1966, he reported the preparation of artificial cells containing magnetic material for targeting the movements of these artificial cells [15]. Later in 1976, he reported the preparation of biodegradable artificial cell membranes using polylactide [16]. This has since been extended by others using polylactic-co-glycolic acid.

Superparamagnetic Fe_3_O_4_ nanoparticles with core dimensions of about 10 to 20 nm exhibit superparamagnetic behavior [17,18]. They have attracted great attention in many fields, such as the development of rare earth elements such as magnetite nanoparticles [19], the use of UV-stable perovskite solar cells [20], and the application of natural polymer magnetic nanocarriers for drug delivery [21]. Due to the excellent biocompatibility and biodegradability of superparamagnetic Fe_3_O_4_ nanoparticles, Fe_3_O_4_-based nanostructured systems could be a potential platform for successfully controlled and targeted drug delivery applications using magnetic hyperthermia. Among magnetic metal nanoparticles, iron oxide nanoparticles (Fe_3_O_4_ NPs) are the only MNPs approved for clinical use by the US Food and Drug Administration [22,23]. Fe_3_O_4_ NPs are non-toxic in vivo because they can be metabolized into oxygen and elemental iron by hydrolytic enzymes, where the iron accumulates in the natural body [24]. The Fe_3_O_4_ nanoparticles as drug carriers can control the drug release pattern, improve the drug bioavailability, and reduce the systemic side effects on healthy tissues [22,25]. In addition, the Fe_3_O_4_ nanoparticles were widely used for thermotherapy in cancer treatment based on the thermal effect of the superparamagnetic Fe_3_O_4_ nanoparticles exposed to irradiation. Superparamagnetic Fe_3_O_4_ nanoparticles have been widely applied to drug delivery for chemotherapeutics and magnetic resonance imaging (MRI) [24]. However, the bare SPIONs (Superparamagnetic Iron Oxide Nanoparticles) are very easy to attract and agglomerate as they are very small in size (nano-dimension), along with the presence of van der Waals force and interparticle magnetic interactions [26]. The surface of Fe_3_O_4_ nanoparticles is prone to modification by conjugating with biochemically active functional groups [27]. The functionalized Fe_3_O_4_ NPs could extend their application to targeted drugs, molecular imaging, enzyme delivery, and cell immobilization [28].

Moreover, the biodegradable and biocompatible artificial cell invented by Chang in 1976 is an alternative solution to solve the above issue [16]. The polymeric shell of artificial cell micro/nanoparticles is a good barrier, not only protecting against the premature release and degradation of drugs, maintaining the sustained release of the drug, but also preventing aggregation from the Fe_3_O_4_ NPs [29,30]. Poly-lactic-co-glycolic acid (PLGA), an FDA and EMA (European Medicines Agency)-approved biodegradable copolymer, is one of the most exploited polymers in the design and formulation of artificial cell nano/micro-drug delivery systems for biomedical applications owing to its biodegradability, biosafety, biocompatibility, and versatility [30,31,32,33]. PLGA can be processed into any shape and size, has excellent water solubility, and allows for a tunable drug release according to its molecular weight and composition ratio [32]. In addition, PLGA can be decomposed into nontoxic compounds (H_2_O and CO_2_) through the Krebs cycle in the human body [34]. At present, more than 20 kinds of PLGA-based biodegradable microspheres and nanoparticles have already been approved by the FDA, many of which are in the stage of R&D or clinical trials [35].

Artificial cell PLGA micro/nanocapsules encapsulating magnetically-responsive Fe_3_O_4_ nanoparticles have been established as multifunctional systems for specific, selective, and performance-enhanced anti-infective [17] and anti-cancer applications [36]. The development of superparamagnetic artificial cells would pave a promising avenue for personalized therapy and precision medicine in the future. To our knowledge, superparamagnetic artificial cell PLGA-drug-Fe_3_O_4_ micro/nanocapsules for targeted cancer therapy have been published discretely in several reviews [34,36,37]. In this review, we discuss the preparation methods of Fe_3_O_4_ NPs and artificial cell PLGA-drug-Fe_3_O_4_ micro/nanocapsules. We shall discuss in detail our approach using emulsion or microfluidizer to prepare suparamagnetic artificial cells of micro and nano dimensions. Then, the medical applications of the superparamagnetic artificial cell PLGA-drug-Fe_3_O_4_ nano/microcapsules for targeted cancer therapy in the last 5 years (2017–2022) were summarized, followed by our insights into the future development of superparamagnetic artificial cell PLGA nano/microcapsules for magnetic targeting cancer treatment.

## 2. Preparation of Fe_3_O_4_ NPs

Fe_3_O_4_ NPs can be synthesized via various methods, including physical, chemical, and biological methods [38]. The method produced can influence particle morphology, size, surface structure, stability, and magnetic properties. And those parameters of Fe_3_O_4_ NPs play an important role in medical applications [39]. Therefore, the selection of the desired method to form the Fe_3_O_4_ NPs shall consider their future biological applications and the differences from the preparation methods of Fe_3_O_4_ NPs. This review will briefly describe the methods of chemical co-precipitation, thermal decomposition, microemulsions, and hydrothermal synthesis, as these preparation methods are simple and convenient and can obtain relatively uniform particles.

### 2.1. Co-Precipitation

Co-precipitation is one of the most popular standard methods for aqueous-phase Fe_3_O_4_ NP synthesis. In simple terms, the generation of SPIONs used aqueous ferrous and ferric salt solutions in a 2:1 proportion in the basic condition, such as NaOH, NH_4_OH, TMAOH, etc. In the reaction process, the reaction mixture was maintained in a pH range of 9 to 11 and at room temperature or higher in an inert environment, for example, Nitrogen or Argon [40,41]. The co-precipitation method has several advantages, including mild reaction conditions, narrow size distribution, facile preparation, and convenient modification of the Fe_3_O_4_ NP surface with other functional groups. Specifically, the particle size can be tuned by adjusting the reaction temperature, the molar ratio of iron salts, solution pH, and ionic strength [42,43]. The production of Fe_3_O_4_ NPs is conducted in the presence of the oxidation/reduction potential of iron salts, which affect the physicochemical properties of the prepared nanoparticles.

### 2.2. Thermal Decomposition

Thermal decomposition can prepare small nanocrystals applied to non-magnetic organometallic precursors in the presence of organic solvents and surfactants at high temperatures ranging from 100 to 350 °C. High-quality SPIONs with sizes of approximately 15 nm can be obtained via this method. The size and morphology of particles mainly depend on factors like ratios and volumes of different precursor molecules and the reaction conditions. Generally, the large Fe_3_O_4_ particle and high saturation magnetization will be obtained over a long reaction time and at a high temperature. For example, Bateer et al. reported that the size of the obtained Fe_3_O_4_ particles increased from 12 nm to 20 nm produced by the thermal decomposition of iron oleate at 350 °C when increasing the reaction time from 40 min to 120 min. Moreover, the saturation magnetization of 12 and 20 nm Fe_3_O_4_ exists at 65.51 and 74.48 emu/g, respectively [44].

### 2.3. Microemulsions

Microemulsions consist of the oil phase, the aqueous phase, and surfactants as stabilizers to form a thermodynamically stable isotropic mixture. There are three main kinds of microemulsions: oil in water, water in oil, and double emulsion. The formation of the particles is confined inside the microemulsion droplets. Thus, the growth of particles is controlled by the size of the microcavity of the droplets. The resulting nanoparticles show small sizes and uniform morphology, good dispersity, and good crystallinity [45,46].

### 2.4. Hydrothermal Synthesis

A simple process for producing Fe_3_O_4_ nanoparticles is hydrothermal synthesis. The iron metal salts, or their oxides, were separated through dehydration and hydrolysis in a solvent under high pressure and temperature conditions. This process required a reactor to maintain a temperature of 150 °C and a pressure of more than 2000 psi [47]. The various physical parameters (temperature, pressure, and reaction time) that affect the distribution and nucleation of the nanoparticles are also taken into account to maintain their good properties [48].

## 3. Methods of Generating Superparamagnetic Artificial Cell PLGA-Drug-Fe_3_O_4_ Micro/Nanocapsules

Various techniques can be used to prepare superparamagnetic artificial cells based on the physicochemical properties of the drug and Fe_3_O_4_NPs micro/nancapsules, such as water-in-oil (W/O) single emulsion [49] and water-in-oil-in-water (W/O/W) double emulsion [50], spray drying [51,52], electrospraying [53,54,55], co-flowing devices [56], and new techniques for microfluidic devices [57,58,59,60]. The processing conditions for producing superparamagnetic artificial cell PLGA micro/nanocapsules play an important role in their final features, such as shape, size distribution, and stability [61]. Here, we describe the most common methods, and they are summarized in Table 1.

### 3.1. Emulsification Method

**Figure 1:**
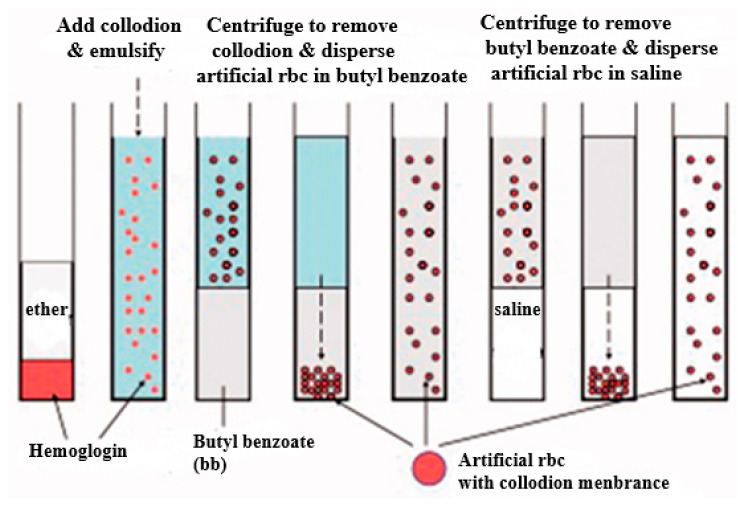
Emulsion method for preparing micro-dimension artificial cells. This image is from the literature. This figure has been taken from ref. [8] with written copyright permission from the publisher, Taylor and Francis.

In 1957, Chang first developed the emulsion method to produce the first artificial cells (Figure 1) [62]. Most nanocapsules and microcapsules are made using the emulsification method because of its simplicity. This method includes the single emulsion method and the double emulsion method [15,16,63,64]. Factors affecting particle size, morphology, encapsulation rate, and drug release include the components of biodegradable polymers, the emulsified concentration of biodegradable polymers, solvent evaporation methods, and surfactants [8,70,71,72]. Different preparation methods can be selected based on the different physical and chemical properties of the encapsulated drug molecules. Most hydrophobic drugs and hydrophobic Fe_3_O_4_ NPs (such as OA-Fe_3_O_4_ NPs) were encapsulated in the PLGA micro/nanocapsules via the single emulsion method of oil in water (O/W) due to the fact that the water-insoluble drug can be well dissolved in the organic solvent to form a homogeneous solution as an oil phase during the emulsified process [49]. In this process, the polymeric solution consisted of a polymer and the desired Fe_3_O_4_ NP in DCM or THF. The resulting mixture solution is then emulsified in a continuous aqueous phase (PVA%) to produce microdroplets/nanocapsules under mechanical stirring or sonication. The organic solvent was removed by evaporating the organic solvent to achieve solidification and the formation of the rude microspheres. After that, the pure microspheres or nanoparticles were obtained by purification with suitable filtration and drying [73]. For hydrophilic drugs, the encapsulation efficiency is lower in a single emulsion, attributed to the fact that the water-soluble compounds could rapidly dissociate into the continuous aqueous phase, leading to much drug loss. To enhance the loading level of hydrophilic drug molecules, the hydrophilic drugs can be encapsulated into the PLGA micro/nanoparticles by the double emulsion method of oil in water in oil (O/W/O) or water in oil in water (W/O/W) [16,74,75]. Generally, the alternative for hydrophilic drugs is to transform the drug to a hydrophobic state or use a mixture in the presence of a stabilizer [75]. Moreover, water-soluble bioactive substances like peptides, proteins, sugar, and vaccines were also encapsulated into the PLGA microcapsules, usually using the water-in-oil-in-water (W/O/W) emulsion method [16].

The emulsion method can conveniently obtain large-scale magnetic PLGA nano/microcapsules in one batch. However, much larger undesired particles are present in the sample, and this method usually obtains polydisperse magnetic PLGA nano/microcapsules with an uncontrollable size distribution [76]. In addition, this approach has other drawbacks, such as residual organic solvents in the prepared nano/microcapsules and surfactant residues on the surface of the prepared nano/microcapsules. Moreover, the drug loading efficiency and particle size of microcapsules could not be precisely controlled by manipulating the stirring rate in secondary emulsion preparation, the viscosity of the organic phase, the concentration of polymer used, and osmotic pressure [77].

### 3.2. Spray Drying

The spray drying technique is ideal for encapsulating heat-sensitive substances into the PLGA nano/microcapsules. Schliehe et al. used the spray-drying method to prepare the magnetic microcapsules [52]. The removal of organic solvents by spraying the emulsion into a hot atmosphere can be used to encapsulate biomolecules such as proteins or peptides, plasmid DNA, and small chemically active molecules, including hydrophilic and hydrophobic drugs [65]. However, this method still has several drawbacks. Some heat-sensitive proteins will lose their activity and native structure in the hot, dry air during the spray-drying process. The laboratory setups usually obtain a low yield of production because products stick to the walls of the drying chamber [78].

### 3.3. Electrospray

Electrospray is particularly suited to generating a narrow-sized distribution of particles from tens of nanometers to hundreds of micrometers [79]. Faramarzi et al. produce PLGA particles containing high magnetite nanoparticles using the electrospray method [55]. In the industry, electrospray is employed to produce biodegradable polymeric encapsulations due to its advantages, including versatility, simplicity, low cost of production, high yield of microspheres, and large-scale production [80]. In addition, sensitive substances such as proteins and cells can be loaded into the PLGA nano/microcapsules [66]. The particle size and morphology can be acquired by tuning the experimental voltage and velocity [81]. For this technology, the main problem is low throughput, which limits its large-scale production [82].

### 3.4. Microfluidic Technology

**Figure 2:**
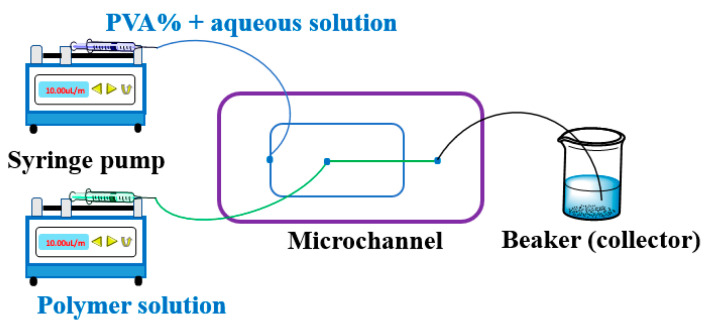
Microfluidics device setup. Two syringe pumps can precisely control the flow rate of both phases, and the flow rate passes through the microchannel of the chip. The resulting microdroplets are collected in the outlet by a beaker as the collector. More information on the microchannel can be obtained from the reference [90,91,92].

Microfluidic technology is a relatedly new strategy for the generation of highly controlled and uniform microspheres [83]. The better formation of micro/nanoparticles can be achieved in a microchannel compared to the conventional method. The microfluidic device consists of a microchip, flow controller, and collector (Figure 2). Generally, the microfluidic chip for preparing the microdroplets (microparticles) is classified into three types: co-flow capillary, flow-focusing capillary, and a combination of co-flow and flow-focusing capillary. The herringbone chip is efficient for the preparation of nanoparticles [84]. In a microfluidic system, the flow phases usually consist of an aqueous phase and an organic phase. The size and polydispersity of the resulting particles were affected by several parameters, such as flow rate, the concentration of both disperse phase and continuous phase, and the surfactant type [85,86]. The low flow rate produces large particles compared to the high flow rate under identical working conditions. The high concentration of the dispersed phase fabricates small particles as opposed to those produced by a low concentration under identical operating parameters. The microfluidic method for preparing PLGA micro/nanoparticles has several advantages: a narrow size distribution, low polydispersity, and reproducibility by controlling flow rate and concentration from bulk preparations. However, the yield of microspheres prepared by this method is relatively low for further biological investigation. However, a series of parallel designs on the microfluidic platform may be possible to achieve the large-scale microspheres and reproductivity of superparamagnetic artificial cell PLGA-drug-Fe_3_O_4_NPs micro/nanocapsules for their further bioassays and other studies [87,88,89].

### 3.5. Nanoprecipitation Method

The nanoprecipitation method is a commonly used technique for preparing magnetic poly(lactic-co-glycolic acid) (PLGA) nanocapsules [67,68,69]. The magnetic nanoparticles are usually coated with a surfactant to prevent agglomeration and ensure good dispersion in the polymer solution. A solution containing PLGA and magnetic nanoparticles is rapidly injected dropwise into a non-solvent solution, such as water or ethanol, under magnetic stirring. This causes the polymer to rapidly precipitate out of solution and form nanoparticles. The disadvantage of this technique is that it cannot precisely control the particle size and size distribution [93], and it is difficult to incorporate hydrophilic drugs using simple nanoprecipitation techniques [94].

## 4. Superparamagnetic Artificial Cell PLGA-Drug-Fe_3_O_4_ Nanocapsules for Cancer Therapeutics

Nanoparticles (NPs) are frequently applied to drug delivery systems (DDS) as they allow delivery of the appropriate drug amount to the targeted site at the desired time [95]. Successful drug delivery systems require high encapsulation efficiency, reduced systemic clearance, and prolonged half-life circulation of the drug in vivo [96]. Biodegradable polymeric artificial cells have gained more and more attention as drug delivery systems to fight malignant tumors and other diseases [8]. Magnetic drug carriers not only achieve high local drug concentrations and avoid the toxicities on other organs and healthy cells, but they can also avoid the influences on the reticuloendothelial system [97,98]. Superparamagnetic artificial cell PLGA-drug-Fe_3_O_4_ NPs micro/nanocapsules could be a promising strategy as magnetic-targeted drug carriers for cancer therapeutics to realize fixed-point, timing, and quantitative drug release [99,100,101]. In this section, we will summarize the applications of superparamagnetic artificial cell PLGA-drug-Fe_3_O_4_ capsules as anticancer drug carriers in cancer therapy, magnetic hyperthermia, and photothermal therapy.

### 4.1. Superparamagnetic Artificial Cell PLGA-Fe_3_O_4_ Nanocapsules as Cancer-Targeted Therapeutics

#### 4.1.1. Lung Cancer Therapy

Lung cancer is a malignant tumor caused by uncontrolled cell growth in the lung tissue [96]. Nanoparticles can directly deliver drugs to the lung to treat lung diseases, improving the drug treatment effect, reducing systemic toxicity, achieving sustained drug release, and improving patient compliance [102]. Numerous studies have shown that biodegradable polymeric nanocapsules can easily penetrate cell membranes, decreasing the untargeted effects and lysosomal escape following endocytosis, which significantly improves the co-delivery efficiency of chemotherapeutic agents [103,104,105]. Tetrandrine (Tet) [106,107] and doxorubicin (DOX) [108] were encapsulated into magnetic PLGA nanoparticles as nanocarriers to treat lung cancer. Tetrandrine (Tet) can inhibit lung cancer cell proliferation, induce apoptosis [109], and suppress lung cancer growth by destructing the VEGF/HIF-1α/ICAM-1 signaling pathway [110] (Table 2). The magnetic nanocapsule Tet-Fe_3_O_4_-PLGA NPs had a higher anti-proliferation effect than the free drug Tet. This is because Tet-Fe_3_O_4_-PLGA nanocapsules could effectively penetrate the A549 cell membrane and multicellular spheroids. These prepared magnetic nano-vehicles can highly injure lysosomes, leading to apoptosis [107]. Similarly, DOX was loaded onto the magnetic PLGA to fabricate nanocarriers (F/A-PLGA@DOX/SPIO NPs) as a dual probe. Folic acid (FA) and activatable cell-penetrating peptide (ACPP) were used to improve the cancer cells’ targeting abilities. In vivo studies had shown that the F/A-PLGA@DOX/SPIO NPs had better inhibitory effects on tumor growth and fewer organ toxicities compared to free DOX. In vivo MR R2* imaging studies showed that the R2* value of tumors treated with the F/A-PLGA@SPIO NPs and F/A-PLGA@DOX/SPIO NPs could increase significantly at 1 h and 4 h compared to the SPIONPs [108].

Poly(ethylene glycol) (PEG) can escape immune recognition, prevent macrophage attachment, enhance retention time for drug delivery [139,140], and minimize the non-specific uptake in normal tissues [141]. Thus, the magnetic PLGA-PEG nanoparticle was used to fabricate a magnetic nanocarrier to deliver silibinin (Sil) [111], curcumin (Cur) [112], and co-deliver silibinin (Sil) with metformin (Met) [113]. In the nanosystem Sil/Met-PLGA-PEG-Fe_3_O_4_NPs, although the saturation magnetization of this magnetic nanocarrier is low (9 emu/g), they still exhibited superparamagnetic properties. In the cytotoxicity assay, Met/Sil-PLGA-PEG-Fe_3_O_4_ NPs at a concentration of 10.51 μM showed a significant inhibitory effect on the lung cancer cell A549 compared to the free drugs (Met and Sil). With increasing concentrations, the Sil/Met-PLGA-PEG-Fe_3_O_4_ NPs group also exhibited higher toxicity than the free Met and Sil groups. This co-delivery carrier could achieve a higher synergistic therapeutic effect compared to the individual agent and could reduce the toxicity of the single agent. That magnetic nano-vehicle followed dose-dependent and time-dependent manners in the reaction with the A549 cells. Experiments revealed that these magnetic PLGA nanoparticles exhibit an outstanding anti-cancer effect and targeting function [113].

#### 4.1.2. Breast Cancer Therapy

Breast cancer is one of the most common forms of malignant tumors among women worldwide and poses a severe threat to their well-being [142]. Several drugs for breast cancer and Fe_3_O_4_ NPs were co-encapsulated into PLGA or PLGA-PEG shells to form the magnetic nanocapsules to carry the drugs to the breast cancer cells, such as Methotrexate (MTX) [114,115], Doxorubicin (DOX) [116,117], Salvigenin (Sal) [118], Gemcitabine (Gem) [119,120], Paclitaxel (PTX) and Transferrin (Tf) [121], Docetaxel (DTX) [122], Vitamin C (Vc) [123], Olaparib (Olb) [124], and Chlorin E6 (Ce6) [125].

To improve the bioavailability and reduce the toxicity of MTX, the MTX was efficiently encapsulated into the magnetic polymer PLGA to obtain a magnetically guided system for breast cancer. The magnetic MPNP-35 with rough surfaces followed the sustained release mode and showed an apparent anti-proliferation effect on breast cancer cells. The optimized experimental condition achieved a high drug encapsulation efficiency of 84.31% in this study [114].

Similarly, to enhance the therapeutic efficacy of the chemotherapeutic agent paclitaxel (PTX) and its magnetic targeting ability, PTX was co-encapsulated with Tf into a magnetic PLGA nanoparticle to obtain enhanced magnetic nanocomposite (Tf-PLGA-PTX-Fe_3_O_4_NPs). Tf is an iron-binding plasma glycoprotein mediated by cell surface Tf receptors overexpressed in proliferating cancer cells. In vitro results and confocal images showed that, compared to the PLGA-PTX-Fe_3_O_4_NPs, the Tf-PLGA-PTX-Fe_3_O_4_NPs exhibited the highest cytotoxicity on the MCF-7 and U-87 cells due to the fact that Tf could promote cellular uptake and drug accumulation in the tumor cells. Moreover, the cellular uptake efficiency of those magnetic nanoparticles was higher when exposed to an external magnetic field [121].

To improve the activation and release of Vitamin C (Vc) and superparamagnetic iron oxide by specific stimuli in the TME, a magnetic nanoreactor system was designed to incorporate Vc and Fe_3_O_4_ NPs [123]. The magnetic system (PLGA-SPIO&Vc) has excellent colloidal stability in the physiological environment within 7 days. However, PLGA-SPIO&Vc would swell well under acidic conditions (pH 6.5) due to the acidic degradation of the PLGA shell. Vc could be significantly released on exposure to LIFU irradiation, especially in acidic conditions, which can promote the chemodynamic therapy efficacy associated with the Fenton reaction. In the flow cytometry, the highest apoptosis of MDA-MB-231 cells (breast cancer cells) was observed in the Ma/PLGA-SPIO&Vc + LIFU group since LIFU irradiation can enhance the permeability of PLGA-SPIO&Vc into the cell membrane, thus facilitating cell phagocytosis. The PLGA-SPIO&Vc were injected into the tumor mice to investigate their pharmacokinetics and biodistribution. The result was that sufficient accumulation of PLGA-SPIO&Vc could be achieved in tumor tissues.

In the case concerning the synergistic platform, both agents of indocyanine green (ICG) and zoledronic acid (ZOL) were co-encapsulated into the magnetic PLGA nanoparticles to form nanocarriers (ICG/Fe_3_O_4_@PLGA-ZOL). With the ability of ICG and Fe_3_O_4_ to convert light into heat, in vivo experiments showed that ICG/Fe_3_O_4_@PLGA-ZOL NPs had remarkable antitumor effects on breast cancer tibial metastasis and could be accurately located in the medullary cavity of the tibia [126].

Likewise, the natural glucose oxidase (GOx) and superparamagnetic Fe_3_O_4_ nanoparticles were co-loaded into magnetic PLGA nanocapsules to fabricate a sequential nanocatalyst (GOx@PLGA-Fe_3_O_4_). The GOx@PLGA-Fe_3_O_4_ NPs group showed higher cytotoxicity than the GOx or Fe_3_O_4_ NPs groups. This is because the GOx@PLGA-Fe_3_O_4_ NPs can produce highly toxic hydroxyl radicals. Moreover, the GOx@PLGA-Fe_3_O_4_ NPs group generated more cellular ROS compared to other groups. The GOx@PLGA-Fe_3_O_4_ nanoparticles were injected into mice to evaluate their tumor-catalytic therapeutics. The results showed that the 4T1 mammary cancer cell growth in the GOx@PLGA-Fe_3_O_4_+magnetic targeting group was significantly inhibited with a suppression rate of about 87.44%, while the non-magnetic targeting group was approximately 46.73% [127].

Polymeric NPs modified with targeting ligands can increase their intracellular delivery ability and maintain sustained drug release [143]. For example, to improve the targeting ability of magnetic PLGA nanoparticles to the human epidermal growth factor receptor 2 (Her2/neu) overexpressed in breast cancers, Herceptin was grafted onto the PLGA-Fe_3_O_4_NPs surface by chemical reaction. In the FACS and MR molecular imaging experiments, a strong contrast enhancement was observed on the Her2/neu overexpressing cell line compared to the Her2/neu non-expressing cell lines, and the signal intensity of in vivo MR imaging was inversely proportional to the concentration of Herceptin [144]. Similarly, L-arginine-based magnetic nanoparticles were constructed for multiple synergistic tumor therapies. The produced Fe_3_O_4_@PLGA/LA NPs could significantly enhance the therapeutic effect since drugs accumulate more and have prolonged retention at the tumor site under an external magnet. This nanoplatform exhibited synergistic therapeutic effects by combining HIFU-based tumor ablation and nitric oxide (NO) to assist antitumor gas therapy. Experiments showed that this novel strategy had excellent synergistic effects for tumor suppression compared to individual HIFU or gas therapy [145]. The surface of PEI-PLGA nanoparticles also allowed further modification with hyaluronic acid (HA) to actively target the CD44 receptor [124].

Immunotherapy has gradually become a new approach to cancer treatment in recent years. However, the massive immunosuppressive cells and tumor-associated macrophages are unfavorable to the therapeutic effects. To solve the challenge of an immunosuppressive tumor microenvironment (TME), a magnetic nanocarrier (PLGA-ION-R837@M (PIR@M)) was developed to selectively target and polarize the macrophages to boost and pave the way for immunotherapy in breast cancer [130].

#### 4.1.3. Glioblastoma Therapy

Glioblastoma (GBM) is one of the fatal malignant tumors occurring in the central nervous system of the human body [146]. It possesses several adverse features, including rapid proliferation, high invasiveness, extensive infiltration, and hyper-neoangiogenesis, complicating treatment with the current therapies [146]. In comparison to conventional chemical drugs, nanomedicine possesses several advantages, such as crossing biological barriers, high accumulation at the targeted brain tumor site, and low systemic toxicity [147].

Research demonstrated that the oleic-based magnetic PEG-PLGA nanoparticles (OAMNP) could serve as a potential magnetic targeting nanocarrier for glioblastoma treatment [148]. Magnetic RGD-PLGA-PEG NPs wrapped paclitaxel (PTX) to obtain a magnetic drug delivery system (PTX/SPIO-RGD-PLGA-PEG NPs), where RGD could enhance the cellular uptake through specific binding to the αvβ3 receptors expressed on U87MG and HUVEC cells. The cellular internalization of PTX/SPIO-RGD-PLGA-PEG was nearly twofold greater than that of PTX/SPIO-PLGA-PEG, with no significant cytotoxic effect on the U87MG cells at a concentration of about 0.01 ng/mL. Their anti-tumor efficacy was evaluated by injecting the PTX-SPIO-PLGA NPs into the orthotopic glioma-bearing mice. In the PTX-SPIO-PLGA NPs group, the tumor growth and tumor area in mice were significantly smaller than in other groups. In comparison to the magnetic therapy, the accumulation of the prepared NPs in the active + magnetic group and the magnetic group was all higher than that for the other group. More importantly, the 6 doses of PTX/SPIO-NPs (5 mg/kg PTX) had no toxic effects on the liver, kidney, or heart compared to Taxol [128]. Similarly, Doxorubicin (DOX) was loaded into the magnetic PLGA nanoparticles to generate PLGA-DOX-Fe_3_O_4_ NPs for fighting glioma cells. The EC_50_ was around 20 µg/mL for all the synthesized SPION-DOX HPNPs in glioma cell lines U87 and 9L after treatment for 48 h. This nanocarrier could also enhance the T2 contrast in MRI [129].

Lanthanide doping with iron oxide nanoparticles could enhance the T1 contrast in MRI [149]. Gadolinium (Gd) was doped into the iron oxide magnetic core to obtain a complex superparamagnetic core (Gd-Fe_3_O_4_ nanoparticles) to produce Gd-Fe_3_O_4_-Lenalidomide-PEI-PLGA NPs. These synthesized nanocarriers still exhibited the superparamagnetic property in the presence of Gd. As a result, the Gd-Fe_3_O_4_-Lenalidomide-PEI-PLGA NPs can significantly inhibit the proliferation of glioblastoma cells, effectively accumulate drugs in the tumor site, and target mitochondria [150].

#### 4.1.4. Other Cancer Therapies

Besides lung, breast, and glioblastoma cancer, the drug-loaded magnetic PLGA nanocapsules were also used to treat other cancers. For example, silibinin (SLB) was loaded into magnetic PLGA nanoparticles to treat renal cell cancer [131]. Paclitaxel (PTX) was encapsulated into magnetic PLGA nanoparticles to obtain magnetic nano-vehicles (PTX-Fe_3_O_4_-PLGA NPs) for fighting endometrial cancer [132]. Curcumin and verapamil were co-encapsulated into magnetic PLGA nanoparticles for the HepG2 cancer treatment [134]. Mohammed Sedki et al. prepared a hybrid nanocarrier system (SMV- Fe_3_O_4_-PLGA-COOH) for enhancing simvastatin cytotoxic activity against prostate cancer [133]. Curcumin (Cur) was loaded in the magnetic PLGA-PEG-FA nanoparticles to obtain multifunctional nano-niosomes (Cur-Fe_3_O_4_-PLGA-PEG-FA) for the treatment of cervical cancer (the HeLa229 cells) [135].

**Figure 3:**
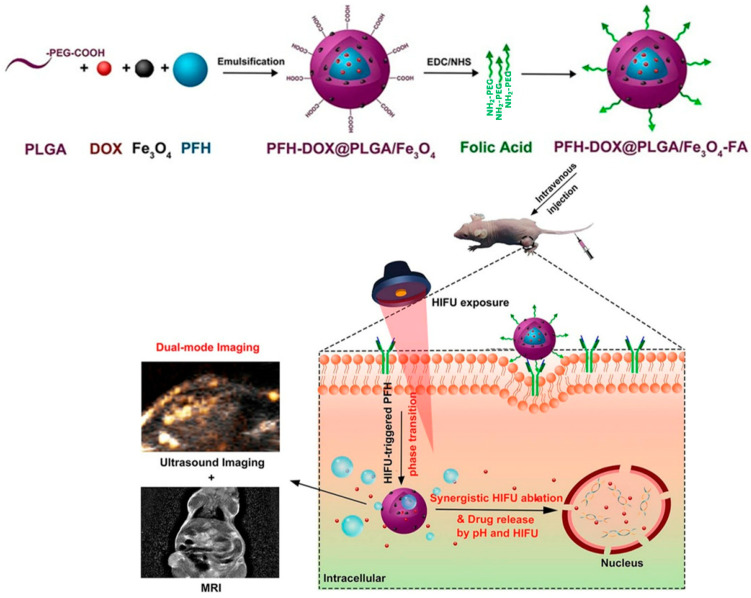
Schematic illustration of the synthesis of PFH/DOX@PLGA/Fe_3_O_4_-FA nanocomposites and corresponding synergistic cancer therapy modals for targeted US/MRI-guided HIFU/chemo. This figure has been taken from ref. [136] with permission from the American Chemical Society, Copyright © 2018.

A synergistic nanosystem with magnetically targeted and temperature-responsive functions was designed to target hepatoma cells (Figure 3). This system (PFH/DOX@PLGA/Fe_3_O_4_–FA) was constructed with biodegradable PLGA, targeting material folate (FA), liquid-gas phase-changeable PFH, and superparamagnetic Fe_3_O_4_ NPs. Both pH and HIFU could control the release of loaded anticancer drugs from the nanocomposites. The animal experiments showed that the PFH/DOX@PLGA/Fe_3_O_4_–FA group had a higher accumulation of magnetic nanoparticles in the tumor site compared to other groups. The PFH/DOX@PLGA/Fe_3_O_4_–FA nanoparticles could enhance the T2 signal contrast. Both contrast-enhanced ultrasound imaging and HIFU ablation efficacy could be improved when PFH/DOX@PLGA/Fe_3_O_4_–FA nanoparticles were treated by high-intensity focused ultrasound. Thus, those prepared nanoparticles could efficiently suppress tumor growth through synergistic chemotherapy and HIFU ablation. Additionally, no toxic effects on normal organs had been observed [136].

The anti-CD133 Ab is the cell surface marker in various cancers, such as brain, prostate, and colorectal cancer. The anti-CD133 Ab was conjugated to superparamagnetic PLGA nanoparticles as a targeting nanocarrier to load Oxaliplatin against CaCo-2 colorectal carcinoma cells [137]. The cRGDa can enhance the targeting ability of the magnetic PLGA nanoparticles to the ovarian cancer cells and also improve ultrasound imaging and magnetic resonance imaging (MRI) in vivo. Thus, the multifunctional tumor-targeted PLGA nanoparticles (PFH/siBIRC5/Fe_3_O_4_@Pt(IV) NPs-cRGD) were prepared to deliver Pt(IV)/siBIRC5 to ovarian cancer cells [138]. Shortly, combining antibodies on the particle surface can enhance the specific affinity of magnetic nanocarriers to tumor cells [140].

### 4.2. Superparamagnetic Artificial Cell PLGA-Fe_3_O_4_ Nanocapsule for Magnetic Hyperthermia and Photothermal Therapy

#### 4.2.1. Superparamagnetic Artificial Cell PLGA-Fe_3_O_4_ Nanocapsules for Magnetic Hyperthermia

Hyperthermia is a promising adjuvant therapy for the treatment of many tumors. Cancer cells have been proven to be more sensitive than normal cells to temperature and pH [151]. Cancer cells would be destroyed at temperatures 41–45 °C by destroying the cell membrane and proteins, which would disturb the nucleic acid synthesis, while healthy cells can still survive [152,153]. Magnetite nanoparticles (MNPs) can generate localized heat under an external magnet and reduce adverse side effects for the normal system. The drug release from superparamagnetic PLGA nanoparticles can be effectively controlled by magnetic induction heating during the treatment of cancer. The glass transition temperature (Tg) of PLGA is around 40 °C [154]. When the temperature is higher than the glass transition temperature of PLGA itself, the polymeric structure will change and accelerate its degradation. Thus, the diffusion and release of the encapsulated drug will be accelerated. Superparamagnetic PLGA nanoparticles can be inductively heated under an external alternating magnetic field (AMF) [155]. These characteristics benefit magnetic hyperthermia ( Table 3) [153,156].

Approximately 5-fluorouracil is a common anticancer drug with undesired side effects on healthy organs and low bioavailability. When 5-fluorouracil was loaded into the magnetic PLGA nanoparticles, these synthesized PLGA-Fe_3_O_4_-5-FU NPs could achieve hyperthermia in the human colon cancer cell line HT-29. The release of 5-FU from PLGA-Fe_3_O_4_-5-FU NPs had no effect at 37 and 43 °C. This property is helpful for the use of cancer treatment. The combination group (PLGA-Fe_3_O_4_-5-FU nanoparticles + hyperthermia) could significantly reduce the survival of tumor cells compared to the 5-FU-PLGA non-hyperthermia nanoparticles group, indicating that the combined method had an excellent inhibitory ability for tumor colony-formation. Moreover, the group treated with 5-FU-loaded nanoparticles could significantly reduce the survival fraction compared to those treated with the free drug 5-FU [157]. These results were because hyperthermia made 5-FU transfer to active forms more rapidly [164], and the cells at 43 °C were more sensitive to hyperthermia than those at physiological temperatures [165].

Similarly, DOX and Fe_3_O_4_ nanoparticles were incorporated into temperature-responsive polymer PLGA nanoparticles to achieve hyperthermia and local heat-triggered chemotherapy for efficient dual cancer treatment [155]. In another study, a thermal-sensitive nano theragnostic agent and phase-shifted magnetic nanoparticles (PMNPs) were combined in a single nanosystem. The PMNPs, which comprise the biocompatible polymer PLGA shell and the liquid core perfluoropentane (PFP), can easily be converted into nano- and/or microbubbles upon heating to physiological temperatures. The PMNPs and MNPs groups could increase the temperature of cell media rapidly compared with the other groups, indicating that PMNPs could act as efficient NIR heat absorbers for hyperthermia therapy (HTT) [158].

Approximately 17-N-allylamino-17-demethoxygeldanamycin (17AAG) was the first Hsp90 inhibitor with broad-spectrum anticancer activities. Combining 17AAG (a Hsp90 inhibitor) and Fe_3_O_4_ magnetic nanoparticles could provide a magnetic hyperthermia strategy for pancreatic cancer treatment [159]. Glycine-arginine-glycine-aspartic acid-serine (GRGDS) can selectively recognize the vβ3/vβ5 integrins overexpressed on the glioblastoma cells. GRGDS conjugated to curcumin-Fe_3_O_4_-PLGA nanoparticles can increase cellular uptake to achieve hyperthermia treatment in glioblastoma cells. These synthesized nanoparticles could generate heat to elevate the local temperature to the hyperthermia treatment degree (~42–45 °C) when exposed to an AC magnetic field for 15 min. GRGDS-Cur-m-PNPs could damage the intact cell membrane when exposed to an RF field [160]. This is because the magnetic field could increase the cellular uptake and permeability of the blood-brain barrier [166].

#### 4.2.2. Superparamagnetic Artificial Cell PLGA-Fe_3_O_4_ Nanocapsule for Photothermal Therapy

Photothermal therapy (PTT) is a less invasive and highly effective therapeutic alternative for cancer treatment that employs photo-absorbing nanoparticles to convert photon energy from near-infrared (NIR) lasers into thermal energy to “burn” the cancer cells.

A triple-modal imaging-guided magnetic nanocarrier (IR780/Fe_3_O_4_@PLGA/PFP/DOX NPs) was produced for breast cancer treatment. Specifically, this complex nano-system consists of near-infrared fluorescence, magnetic resonance, and ultrasound. The samples were irradiated with an 808 nm laser for 5 min in vitro. The temperatures of IR780/Fe_3_O_4_@PLGA/PFP/DOX NPs were 54 °C, and the photothermal conversion efficiency (η) of them (1.0 mg/mL) was 35.7%, showing excellent PTT performance. When combined with laser irradiation, the DOX release from the IR780/Fe_3_O_4_@PLGA/PFP/DOX NPs was as high as 35% within 30 min and 90% within 72 h. Both in vitro and in vivo studies revealed that the prepared magnetic nano-system possesses excellent synergistic performance in magnetic targeting, chemo-photothermal therapy of cancer [117].

Indocyanine green (ICG) is the only NIR agent approved by the U.S. Food and Drug Administration (FDA) for human clinical NIR fluorescence (NIRF) imaging and diagnosis [167]. To overcome its instability in aqueous solutions, rapid blood clearance, and light- and temperature-dependent properties, ICG and pefluoropentane (PFP) were integrated into magnetic PLGA nanoparticles to form dual NIR light-absorbing PFC-based polymeric NPs (Fe_3_O_4_/ICG@PLGA/PFP NPs). Because Fe_3_O_4_ NPs and ICG contribute to NIR light-absorbing agents efficiently converting absorbed light into heat, Fe_3_O_4_/ICG@PLGA/PFP NPs could effectively generate heat when exposed to NIR laser irradiation. When MCF-7 breast cancer cells were treated with Fe_3_O_4_/ICG@PLGA/PFP NPs and irradiated by an NIR laser, the cells were severely damaged. Fe_3_O_4_/ICG@PLGA/PFP NPs were injected in mice with MCF-7 breast tumor cells and could ablate tumor cells under NIR laser irradiation [161].

In another case, ICG and glucose oxidase (GOx) were covalently attached to the surface of the iron oxide nanoparticles to obtain a magnetic photothermal agent (ICGOx@IO). Then this generated magnetic core (ICGOx@IO NPs), DOX, and EGCG ((-)-epigallocatechin-3-gallate) were co-encapsulated into PLGA NPs against melanoma tumors. This complex nanosystem exhibited excellent photothermal efficiency when irradiated for 10 min with an external magnet. The prepared magnetic nanoparticles exhibited significantly higher cytotoxicity than the free DOX and EGCG [162]. Similarly, only ICG was covalently attached to the iron oxide (IO) nanoparticle surface to obtain a magnetic photothermal agent (PIO NPs). Then the PIO NPs, DOX, and Mcl-1-siRNA were co-encapsulated into PLGA-CS NPs to treat breast cancer. In this nanosystem, the siRNAs can overcome the chemoresistance in the breast cells. PIO-DOX-siRNA-PCSCM NPs could maintain good photothermal effects and increase solution temperature under NIR laser irradiation [163].

AS1411 is a specific aptamer to nucleolin expressed in pancreatic cancer cells. The magnetic PLGA-curcumin nanoparticles modified with AS1411 can enhance their specific targeting capability for cancer cells. This prepared nanocomposite could also enhance the photoacoustic (PA) signal contrast (around 900–950 nm). They showed good biocompatibility and low cellular cytotoxicity on the L929 cells compared to SPION alone, attributed to the presence of the AS1411 aptamer. The temperature of the cancer cells treated with the produced magnetic nanoparticles could reach 65 ± 3 °C from 27 °C when exposed to an NIR laser within 5 min, induced photothermal ablation of cancer cells [69].

## 5. Superparamagnetic Artificial Cell PLGA-Drug-Fe_3_O_4_ Microcapsules for Cancer Therapeutics

Along with nanoparticles, micro-dimension microspheres (MPs) are another popular drug delivery system (DDS), as they can achieve targeted treatment and control drug amounts [29,36,142]. The drug release rate from the polymer microspheres could be controlled by optimizing the polymeric coating formulation, such as the molecular weight and the composition of the polymers [168]. The superparamagnetic polymeric microspheres (MMS) usually consist of a magnetic core (Fe_3_O_4_ NPs) and polymeric shells, including PLA, PLGA, and PLGA-PEG [169]. The microsphere size usually ranges from 10 to 200 μm in the clinic. Outside of these ranges, the smaller microspheres will be engulfed by the immune cells, and the larger microspheres will cause inflammation [170]. The PLGA-embedded drug micro-carriers have exhibited outstanding sustained release characteristics and achieved local accumulations in the desired tumor sites. Several drugs were successfully encapsulated into the magnetic PLGA microcapsules to form the micro-carriers, such as 5-fluorouracil (5-FLU) [171], curcumin (CUR) [49,172], camptothecin (CPT) [173,174,175], paclitaxel (PTX) [176], doxorubicin (DOX) [177], and vincristine (Vin) [54] (Table 4 ). To achieve a targeted therapy and reduce the surrounding healthy cell damage caused by the AMF irradiation, the Fe_3_O_4_ nanoparticles and PTX were co-loaded into PLGA microspheres to achieve magnetic navigation [176].

To solve the limitations on solid tumor treatments, including the limited penetration of anti-cancer drugs in solid tumors, the side effects, the development of drug resistance, and the poor cellular uptake of common cancer therapies, the chemical drugs DOX and Vin were co-encapsulated into magnetic PLGA microspheres. The magnetic saturation of the PLGA-Fe_3_O_4_ microspheres (0.6–1.4 emu/g) is much lower than that of free Fe_3_O_4_ NPs (70.5 emu/g). This is because the content of Fe_3_O_4_ in the magnetic microspheres is about 2%. Despite this, those magnetic PLGA microspheres still exhibited superparamagnetic properties. The entrapment efficiency of the drug in the shell is greater than that inside the microsphere. This drug distribution would affect the drug release pattern and the release rate. However, those generated magnetic microspheres followed a sustained release profile. Especially, Fe_3_O_4_@(P + V)/D microspheres could significantly inhibit the growth of osteosarcoma saos-2 cells at a concentration of 100 μg/mL within 48 h with only 14.9% cell viability, indicating that they had an excellent anti-tumor effect [54].

Magnetic microspheres can generate heat by magnetothermal conversion when exposed to an alternating magnetic field [175]. The drug diffusion rate and the polymer degradation rate can be changed when the local temperature is increased by magnetothermal conversion, resulting in an influence on drug release kinetics [178]. A magnetic drug microcarrier was made of magnetic nanoparticles (MNP), camptothecin (CPT), and a polymeric matrix PLGA shell. The drug burst release occurred at 37 and 43 °C in the first hour, followed by a plateau phase. The magnetic PLGA microspheres obtained had an excellent heating performance of 161 W/gM. When applying a magnetic field, the magnetic microspheres were heated to 44 °C within one hour, and the drug from the prepared microspheres released about 50% more than that at 37 °C [175].

Reactive oxygen species (ROS) can damage biological macromolecules such as nucleic acids [179]. Hydrogen peroxide (H_2_O_2_) is one source of ROS, which plays a crucial role in cell signaling pathways. The toxic effects of excess ROS will cause a range of pathologies, such as cancer, aging, and diabetes [180]. A ROS-sensitive magnetic micro-carrier was fabricated to deliver the antitumor drug camptothecin (CPT). This magnetic micro-carrier consisted of ROS-sensitive tween 80-coated Fe_3_O_4_ NPs, CPT, and a biodegradable PLGA-PVA layer. The addition of PVA increased the swelling properties of the CPT-MMSs, which further improved a significant drug encapsulation efficiency of ∼92% and had a higher drug content of ∼15%. CPT-MMSs showed a significantly higher releasing efficiency (90%) in the presence of ROS (H_2_O_2_) than in the absence of H_2_O_2_ (40%) at pH 5.4. CPT-MMS-4 exhibited the highest cytotoxic effect on HeLa cells (IC_50_ = 0.9 ± 0.38 μg/mL) compared to free CPT (5.2 ± 0.23 μg/mL) and Tween 80-MIONs (19.5 ± 0.98 μg/mL) [174].

Nanomedicines combining chemotherapy and photothermal therapy can compensate for the deficiencies of the traditional approaches to osteosarcoma treatment. When individual photothermal therapy is insufficient to ablate the tumor cells completely, the residual tumor cells can also be effectively killed by increasing the drug release using the rising temperature [181]. Multifunctional magnetic microcapsules PB@(Fe_3_O_4_@PEG-PLGA) MCs and (PB+DOX)@(Fe_3_O_4_@PEG-PLGA)MCs were developed for osteosarcoma therapy. They possessed magnetic targeting ability and chemo-photothermal therapeutic efficiency. Simultaneous NIR laser irradiation and magnetic field could enhance the drug release rate from PB@(Fe_3_O_4_@PEG-PLGA) MCs and (PB+DOX)@(Fe_3_O_4_@PEG-PLGA)MCs. PB@(Fe_3_O_4_@PEG-PLGA) MCs could effectively target the tumor tissue against the invasion of osteosarcoma and the alleviation of osteolytic lesions [177].

## 6. Conclusions

In this review, we have introduced the preparation methods of Fe_3_O_4_ NPs and presented the drug and superparamagnetic Fe_3_O_4_ NPs encapsulated into the artificial cells as PLGA micro/nanocapsules. More importantly, we summarized the applications of superparamagnetic artificial cells (PLGA-Fe_3_O_4_) micro/nanocapsules in cancer-targeted therapy. At present, superparamagnetic artificial cell PLGA-Fe_3_O_4_ micro/nanocapsules have many advantages, such as high drug loading and controllable release behavior, magnetic targeting, and combination with other therapy methods. Moreover, superparamagnetic artificial cell PLGA-Fe_3_O_4_ micro/nanocapsules can also integrate multiple drugs as multifunctional platforms to treat cancer.

The size and distribution of superparamagnetic artificial cells PLGA-Fe_3_O_4_ micro/nanocapsules will affect the drug loading capacity and release rate of the drugs from artificial cells PLGA micro/nanocapsules, biodistribution, and bioavailability together. Therefore, to obtain uniform superparamagnetic artificial cells, PLGA-Fe_3_O_4_-drug micro/nanocapsules are essential for their applications in anti-tumor treatment. However, many current studies are only focused on some tumor models and small active molecules, and there are still many difficulties in achieving clinical applications, such as how to overcome the cytotoxicity of materials, prepare uniform particles to increase the uptake of cells, and achieve precise control and personalized treatment. Therefore, we can extrapolate the application of artificial PLGA micro/nanocapsules (Fe_3_O_4_NPs) in the delivery of the gene, cell, stem cell, oxygen carrier, NO donor, or other complex biological substances. We could extend the simple core-shell structure of artificial cell PLGA-Fe_3_O_4_ nano/microcapsules to multi-compartments to allow the integration of multiple active substances in a single carrier to complete synergistic delivery for cancer treatment. Moreover, we could also pay more attention to the development of preparation technology for complex magnetic PLGA-drug artificial cells and the large-scale production and reproductivity of prepared technology for artificial cell PLGA-Fe_3_O_4_-drug micro/nanocapsules in the future.

## Figures and Tables

**Table 1:** The summarizations of the different techniques for supramagnetic artificial cells production.

Techniques	Applications and the Corresponding Reference		Main Advantages	Main Disadvantages
	Nanocapsules	Microcapsules		
Emulsification	[62,63,64]	[49]	conveniently obtaining large-scale PLGA nano/microcapsules in one batch	residual organic solvents in the prepared nano/microcapsules cannot be precisely controlled by particle size
Spray drying	[52,65]	[51,52]	encapsulating heat-sensitive substances	some heat-sensitive proteins will lose their activity and native structure, resulting in a low yield of production
Microfluidic technology	[57,58,59,60]	[57,58,59,60]	a narrow size distribution, low polydispersity, and good reproducibility	low yield of microspheres
Electrospray	[53,54,55]	[66]	generating narrow size distribution of particles, encapsulating sensitive substances	low throughput
Nanoprecipitation	[67,68,69]		conveniently obtaining large-scale PLGA nanocapsules in one batch	can not precisely control the particle size and size distribution, making it difficult to incorporate hydrophilic drugs

**Table 2:** The summarizations of the superparamagnetic artificial cell PLGA-drug-Fe_3_O_4_ nanocapsules for cancer therapeutics.

Entry	Cancer Therapeutics	The Encapsulated Drug and the Corresponding Reference
1	Lung cancer	Tetrandrine [106,107], Doxorubicin [108], Silibinin [111], Curcumin [112], Silibinin + Metformin [113]
2	Breast cancer	Methotrexate [114,115], Doxorubicin [116,117], Salvigenin [118], Gemcitabine [119,120], Paclitaxel + Transferrin [121], Docetaxel [122], Vitamin C [123], Olaparib [124], Chlorin E6 [125], Indocyanine green + Zoledronic acid [126], Glucose oxidase [127]
3	Glioblastoma	Paclitaxel [128], Doxorubicin [129], Lenalidomide [130]
4	Renal cell cancer	Silibinin [131]
5	Endometrial cancer	Paclitaxel [132]
6	Prostate cancer	Simvastatin [133]
7	HepG2 cancer	Curcumin and Verapamil [134]
8	Cervical cancer	Curcumin [135]
9	Hepatoma cells	Doxorubicin [136]
10	CaCo-2 colorectal carcinoma cells	Oxaliplatin [137]
12	Ovarian cancer cells	Pt(IV)/siBIRC5 [138]

**Table 3:** The summarizations of the superparamagnetic artificial cell PLGA-Fe_3_O_4_ nanocapsules for magnetic hyperthermia and photothermal therapy.

Magnetic Hyperthermia and Photothermal Therapy	Cancer Theraeutics	Nanosystem and the Corresponding Reference
	CT26 colon cancer cells	PLGA-Fe_3_O_4_-doxorubicin [155]
	Human colon cancer	PLGA-Fe_3_O_4_-5-fluorouracil [157]
	MCF-7 cells	PLGA-Fe_3_O_4_-perfluoropentane [158]
	Pancreatic cancer	PLGA-Fe_3_O_4_-17-N-allylamino-17-demethoxygeldanamycin [159]
	Glioblastoma cells	Curcumin-Fe_3_O_4_-PLGA-GRGDS [160]
Photothermal therapy	L929 cells	PLGA-curcumin-AS1411 [69]
	Breast cancer	IR780-Fe_3_O_4-_PLGA-PFP-DOX [117]
	MCF-7 cells	Fe_3_O_4-_ICG-PLGA-PFP [161]
	Melanoma tumor	ICGOx-Fe_3_O_4_-PLGA-DOX-EGCG [162]
	Breast cancer	PIO-Dox-siRNA-PCSCM [163]

**Table 4:** The summarizations of the superparamagnetic artificial cell PLGA-drug-Fe_3_O_4_ microcapsule for cancer therapeutics.

Entry	Cancer Therapeutics	The Loaded Drug and the Corresponding Reference
1	Osteosarcoma saos-2 cells	Doxorubicin + Vincristine [54]
2	Solid tumors	5-fluorouracil [171]
3	HeLa cell lines, A549 and HeLa S3 cancer cell lines	Curcumin [49,172]
4	HeLa cells	Camptothecin [173,174,175]
5	HepG2 cells and HL7702 cells	Paclitaxel [176]
6	Osteosarcoma therapy	Doxorubicin [177]

## Data Availability

Data are contained within the article.
